# Marine temperatures underestimated for past greenhouse climate

**DOI:** 10.1038/s41598-021-98528-1

**Published:** 2021-09-27

**Authors:** Madeleine L. Vickers, Stefano M. Bernasconi, Clemens V. Ullmann, Stefanie Lode, Nathan Looser, Luiz Grafulha Morales, Gregory D. Price, Philip R. Wilby, Iben Winther Hougård, Stephen P. Hesselbo, Christoph Korte

**Affiliations:** 1 Faculty of Science, Geology Section, University of Copenhagen, Øster Voldgade 10, 1350 Copenhagen K, Denmark; 2 Geologisches Institut, Dep. Erdwissenschaften, ETH Zürich, Sonneggstrasse 5, 8092 Zürich, Switzerland; 3 Department of Earth Sciences, University of Oxford, South Parks Road, Oxford, OX1 3AN UK; 4 Camborne School of Mines, University of Exeter, Penryn Campus, Penryn, TR10 9FE Cornwall UK; 5 Department of Petrology and Economic Geology, Geological Survey of Denmark and Greenland, Øster Voldgade 10, 1350 Copenhagen K, Denmark; 6 Scientific Centre for Optical and Electron Microscopy (ScopeM), ETH Zürich, Otto-Stern-Weg 3, 8093 Zürich, Switzerland; 7 School of Geography, Earth and Environmental Sciences, Plymouth University, Drake Circus, Plymouth, PL4 8AA UK; 8 British Geological Survey, Keyworth, Nottingham, NG12 5GG UK; 9 School of Geography, Geology and the Environment, University of Leicester, University Road, Leicester, LE1 7RH UK

**Keywords:** Climate sciences, Palaeoceanography, Palaeoclimate, Geochemistry

## Abstract

Understanding the Earth’s climate system during past periods of high atmospheric CO_2_ is crucial for forecasting climate change under anthropogenically-elevated CO_2_. The Mesozoic Era is believed to have coincided with a long-term Greenhouse climate, and many of our temperature reconstructions come from stable isotopes of marine biotic calcite, in particular from belemnites, an extinct group of molluscs with carbonate hard-parts. Yet, temperatures reconstructed from the oxygen isotope composition of belemnites are consistently colder than those derived from other temperature proxies, leading to large uncertainties around Mesozoic sea temperatures. Here we apply clumped isotope palaeothermometry to two distinct carbonate phases from exceptionally well-preserved belemnites in order to constrain their living habitat, and improve temperature reconstructions based on stable oxygen isotopes. We show that belemnites precipitated both aragonite and calcite in warm, open ocean surface waters, and demonstrate how previous low estimates of belemnite calcification temperatures has led to widespread underestimation of Mesozoic sea temperatures by ca. 12 °C, raising estimates of some of the lowest temperature estimates for the Jurassic period to values which approach modern mid-latitude sea surface temperatures. Our findings enable accurate recalculation of global Mesozoic belemnite temperatures, and will thus improve our understanding of Greenhouse climate dynamics.

## Introduction

Accurately reconstructing the Earth’s climate through geological time is important for understanding Earth system feedbacks and for forecasting future climate change^1^. In particular, past periods of highly elevated atmospheric CO_2_, where Greenhouse conditions prevailed, may provide important insights into climate processes operating under anthropogenically elevated CO_2_. The middle and late Mesozoic (Jurassic and Cretaceous; 201–66 Ma) saw such warm-climate processes, wherein polar temperatures were so high that polar ice-caps were absent most of the time^2^. Reconstructing sea and land temperatures during this interval remains challenging. Climate proxies applied to younger sediments (e.g. ice-core and tree-ring records, alkenone biomarkers) are unavailable this far back in time, and many Mesozoic sediments have undergone significant post-depositional thermal and diagenetic alteration, modifying the original composition of fossils. Nonetheless, biogenic calcite from organisms such as brachiopods, bivalves, and particularly belemnites is frequently preserved in such sediments, enabling the application of oxygen isotope thermometry for sea-water temperature reconstructions. Belemnites, emerging in the earliest Jurassic, were squid-like cephalopods (Mollusca) that built their internal skeleton from calcite and aragonite, and went extinct at the end of the Cretaceous. Their ubiquity within Jurassic and Cretaceous seas, and the high preservation potential of their low-Mg calcite skeleton (rostra), make them a favoured target for temperature reconstructions via oxygen isotope thermometry^3–12^. However, two major limitations to this method have led to uncertainties in Mesozoic temperature reconstructions. The first is that the oxygen isotope composition of skeletal carbonates in marine systems vary as a function of both the ambient temperature and oxygen isotope composition of the seawater (δ^18^O_sw_). Mesozoic δ^18^O_sw_ cannot be measured directly, and is usually assumed to be the average value for seawater in an ice-free world (-1‰ SMOW^13^) ; yet the δ^18^O_sw_ value at a given locality and depth in the ocean may deviate from this average by as much as 4‰^14,15^. The second limitation is that numerous equations have been determined for the relationship between temperature and δ^18^O in different calcite types, e.g. molluscan calcite^16–18^, brachiopod calcite^19,20^, foraminifera^21,22^, barnacle calcite^23^, meteoric speleothems and cements^24–28^, and synthetic calcite^29,30^. It is not known if belemnites fractionated ^18^O to the same extent as modern biotic carbonates, and therefore which equation, if any, is appropriate for belemnite calcite. The molluscan equation of Anderson and Arthur^18^ or the general synthetic calcite equation of Kim and O'Neil^30^ are most commonly used for belemnite calcite temperature reconstructions, yet it is observed that belemnites often give similar, or, in many cases, lower temperatures (i.e. higher δ^18^O_calcite_) than co-occurring benthic biotic calcites^3–6,8,11,31^. It has been hypothesised that belemnites may have been migratory to areas of colder and/or isotopically distinct waters^31,32^, or that early diagenetic infill (i.e. as the belemnite skeleton lay on the seafloor) biases belemnite calcite to colder temperatures^33^, although this is disputed by other geochemical studies^34,35^. Furthermore, clumped isotope studies comparing the (visibly) porous apical area to the (less visibly porous) rest of the belemnite rostrum return colder temperatures for the apical area, contradicting a diagenetic cause^35^. Biomarker-based (TEX_86_) temperature reconstructions for shallow-buried (i.e. especially immature) Mesozoic sections similarly give much warmer temperatures than belemnite rostra^7,36^. As the TEX_86_ method is thought to record sea surface temperatures only, it was argued that the belemnites may have been nektonic or nektobenthic, living mostly below the thermocline in colder waters than the surface^7,31^. Yet this does not explain how belemnites may give colder temperatures than co-occurring benthic biotic calcites^3,6,8,11,31^. Clumped isotope thermometry reconstructs belemnite calcite temperatures closer to sea surface temperatures than bottom water temperatures, considerably warmer than oxygen isotope thermometry^35,37–41^. This suggests that either belemnites inhabited highly saline waters (with δ^18^O_sw_ elevated by evaporation), or that the standard calcite thermometry equations are inappropriate for belemnite calcite^35,37–41^.

**Figure 1:**
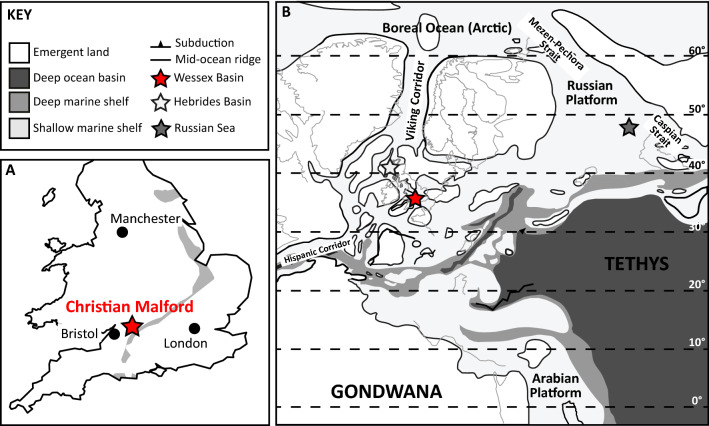
(**A**) Sampling location (Christian Malford) in the U.K., denoted by star, with the outcrop of Callovian sediments indicated by grey band, after Price et al.^42^. (**B**) Palaeogeographic reconstruction of the Tethyan Realm during the Middle Jurassic, after Dera et al.^63^.

Here we present new clumped isotope data derived from co-occurring calcite and aragonite in individual exceptionally well preserved belemnites (*Cylindroteuthis*)^42^ from the Callovian-aged Christian Malford Lagerstätte (Fig. 1). These samples are compared to other (non-belemnite) aragonites from the same horizon in order to evaluate the putative temperature record based upon the oxygen isotopic composition of well-preserved marine fossils, and thereby to resolve which oxygen thermometry equation is most appropriate for belemnite rostra in Mesozoic paleoclimate studies. In doing so, we highlight significant systematic underestimations of global sea temperature in Mesozoic reconstructions. Our warm temperatures are more in line with the geographical distributions of temperature-sensitive fossil flora and fauna, as well as the results of climate models with increased CO_2_ levels, and demonstrate that at least several common belemnite genera lived neither deep in the water column nor in hypersaline conditions.

## Results

### Preservation

The Christian Malford Lagerstätte is known for its exceptional preservation of biomaterials in fossil marine organisms^42–44^. The host sediments have experienced minimal burial and thermal maturation as indicated by the immaturity of their organic matter^43,45,46^. Powder X-Ray diffraction (PXRD) confirms the preservation of original aragonite (Supplementary Fig. S3), which is uncommon in sediments this old due to the metastability of aragonite at Earth surface temperatures and pressures^47^, and further confirms the extremely low thermal maturity. Belemnite rostral calcite shows extremely low Mn/Ca and Fe/Ca values (Supplementary Fig. S12), in agreement with Price et al.^42^, and electron backscatter diffraction (EBSD) and scanning electron microscope (SEM) electron dispersive spectra (EDS) element maps show that no perceptible diagenetic alteration occurred in the non-apical areas of the belemnite rostra (Supplementary Figs. S7, S8, S9, S10, S11).

Whilst the aragonite and calcite appear texturally and geochemically pristine, studies have shown that clumped isotope (Δ_47_) temperatures may be increased by re-ordering of the ^13^C-^18^O bonds (‘solid state reordering’), a process by which no minor element or visible change, even at the microscopic level, occurs in the carbonate^48–50^. For calcite formed at ambient temperatures, this re-ordering may occur where the samples experience temperatures above 80–100 °C over geological timescales (millions of years)^49–51^. Aragonite is much more prone to alteration and its reordering kinetics are much faster than those of calcite^48^; e.g. Ritter et al.^52^ showed that such reordering may occur after 20 weeks of the aragonite being held at 100 °C. However, for the Callovian sediments at Christian Malford, burial estimates, maturity indices and diagenetic carbonate clumped isotope data suggest that such temperatures were not reached^43,45,46^, negating this effect. In combination, these conditions provide a unique opportunity to determine the accuracy with which belemnite rostra record original calcification temperatures and can be relied upon to faithfully record ambient marine conditions in the Mesozoic. If aragonite and calcite from the same organism yield the same temperature, we can be very confident that they represent true original temperatures, as any alteration would preferentially disrupt the values for the aragonite over the calcite.

### Clumped isotope palaeothermometry

**Figure 2:**
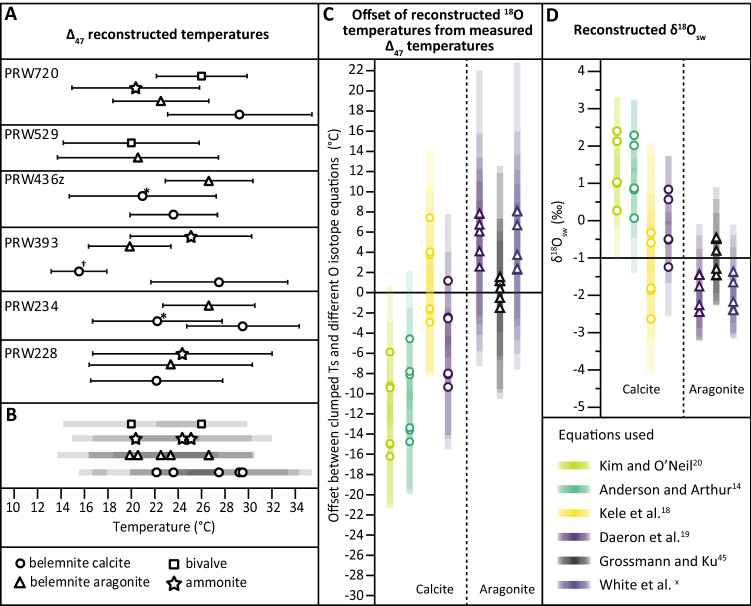
(**A**) Reconstructed temperatures from clumped isotopes (each based on 10 or more replicates) for all samples analysed (all from the Phaeinum subzone), displayed by type (rostrum, phragmocone, ammonite and bivalve) and with accompanying samples from the same block. Error bars indicate the 95% confidence interval. † = sample from apical area, including early diagenetic infill. * = diagenetic sparry calcite from phragmocone chamber spaces. The calcites marked †* are not included in any following analyses of the data (e.g. Figure 2B, C or Fig. 3). (**B**) All samples grouped together to demonstrate the range spanned over this single subzone, not including the samples marked †*. (**C**) Offset of reconstructed ^18^O temperatures (for the different equations) from measured clumped isotope temperatures. Error envelope = maximum possible offset between clumped and stable temperatures based on the 95% CL level for clumped isotope temperatures and the SD for measured oxygen isotopes.

Clumped isotope compositions for the analysed calcites and aragonites range from 0.580 to 0.609 (I-CDES^53^; excluding separated diagenetic calcites from the apical area and spar cements), which correspond to temperatures ranging from 20.0 to 29.5 °C based on the Anderson et al.^54^ equation. Anderson et al.^54^ found that inorganic, and most biogenic, carbonates (including aragonite) show the same temperature dependence; thus, their equation can confidently be applied to reconstruct palaeotemperatures for aragonites and calcites. Reconstructed aragonite and calcite Δ_47_ palaeotemperatures from the same belemnite are consistently within error of each other, and are in agreement with other biotic aragonites from the sample (Fig. 2A). This supports that the observed Δ_47_ temperatures are pristine. This is therefore the first study that provides seawater temperatures and oxygen isotope compositions which are proven to be unaffected by reordering.

## Discussion

This study, along with other clumped isotope studies of belemnites^35,37–41^ finds that belemnites grew in much warmer (i.e. near-surface) waters than previous stable isotope studies have suggested^3–12^. Clumped isotope records show that belemnites record warmer temperatures than early (seafloor) diagenetic cements^35^ (Fig. 2A), consistent with recent palaeontological work that shows that some belemnites lived in the top 200 m of the water column^55^.

Traditional stable oxygen isotope thermometry (e.g. Anderson and Arthur^18^ equation, assumed δ^18^O_sw_ = − 1 ‰ for an ice-free world^13^) consistently returns temperatures for rostral calcite that are, on average, 9 °C colder than for phragmocone aragonite (using the Grossman and Ku^56^ aragonite equation) from the same individual belemnite (Fig. 2). Whilst this phenomenon has previously been observed^42^, it was not possible to determine if the aragonite temperatures were too warm or if the Anderson and Arthur^18^ equation gives temperatures that are too cold. It is believed that belemnite calcite precipitated near Δ_47_ isotopic equilibrium^41,57^, and the consistency between belemnite calcite and aragonite clumped isotope temperatures (Fig. 2) suggests that belemnite aragonite also does not exhibit strong kinetic disequilibrium effects with respect to Δ_47_, despite this phenomenon having been observed in modern cephalopod aragonite^58,59^. In modern cephalopods, disequilibrium effects lower Δ_47_ values, i.e. yielding temperatures that are warmer than the true growth temperature, by as much as 8 °C^58,59^, yet belemnite aragonite in this study does not yield warmer temperatures than the rostral calcite (Fig. 2A). Thus we proceed under the assumption that both calcite and aragonite from belemnites precipitated in Δ_47_ equilibrium.

Several more recent studies present equations determined for natural calcite δ^18^O grown in equilibrium. Two studies present equations derived from very slow-growing subaqueous calcites^26,28^. Daëron et al.^28^, being the most recent, is given below (Eq. 1). Another stable isotope equation derived for fast-growing travertines, is believed to reflect (near) equilibrium conditions^27^, yet differs from Daeron et al.^28^ in having a slightly steeper slope (Eq. 2):

1 $$1000ln\alpha = 27.57*\left( \frac{1000}{T} \right) - 29.13$$

2 $$1000ln\alpha = 20*\left( \frac{1000}{T} \right) - 36$$

where α is the calcite/water oxygen-18 fractionation factor and *T* is the absolute temperature in Kelvin.

We do not know the δ^18^O aragonite-water fractionation for belemnite phragmocone, yet for modern aragonitic cephalopods this effect is negligible, and closely approximates both the equilibrium calibration of Daëron et al.^28^, and the biogenic calibrations of White et al.^60^ and Grossman and Ku^56,58,59^. However, calcites may show near-equilibrium Δ_47_ yet far-from-equilibrium δ^18^O^61^, and as there are no studies on calcifying cephalopods, we do not know if the biotically-mediated precipitation of rostral calcite may have been fractionated with respect to seawater. As clumped isotope thermometry gives both the temperature and δ^18^O_carbonate_, these can be used together to back-calculate the δ^18^O_sw_, using one of the stable isotope equations. It is hard to reconcile two carbonate phases from the same organism growing in different δ^18^O_sw_; thus, we can select the equations that yield the most similar δ^18^O_sw_ for belemnite calcite and aragonite pairs (Fig. 2D). The closest matches are between the Kele et al.^27^ equation for the rostra and any of the aragonite equations^28,56,60^ (Fig. 2D) for the phragmocone; closely followed by Daëron et al.^28^ for the rostral calcite with Grossman and Ku^56^ for aragonite (Fig. 2D and Supplementary data). Interestingly, applying Daëron et al.^28^ to both calcite and aragonite gives a significant offset between reconstructed δ^18^O_sw_—the aragonite is c. 1.5 ‰ lighter than the calculated calcite average δ^18^O_sw_ (Fig. 2D and Supplementary data). The Kele et al.^27^ equation, derived for fast-growing travertines, is believed to reflect (near) equilibrium conditions, yet differs from Daëron et al.^28^ in having a slightly steeper slope (Eq. 2). Kele et al.^27^ could not explain the different slope of the travertine curve by any physical or chemical parameter (including growth kinetics). For belemnite calcite, the close fit to the Kele et al.^27^ equation may imply that some biotically-driven fractionation of ^18^O occurred during precipitation of the calcite from the belemnite body fluid (since the aragonite was precipitated from body fluid in equilibrium with seawater^28^).

**Figure 3:**
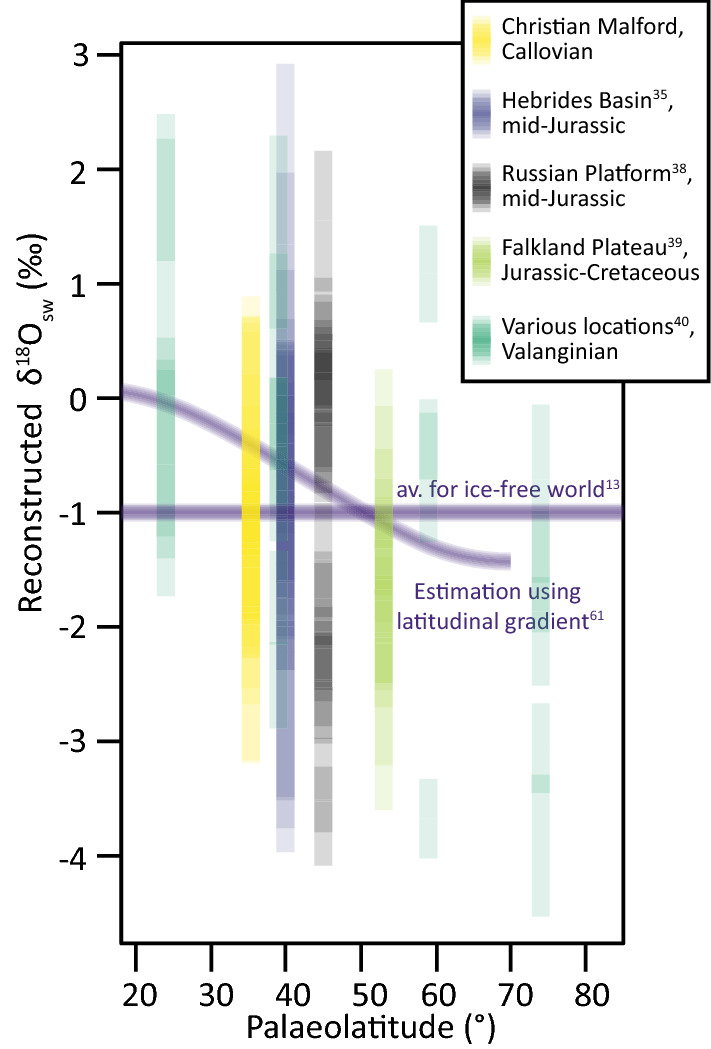
Reconstructed δ^18^O_sw_ based on clumped isotope temperatures using the Kele et al.^27^ equation according to palaeolatitude, compared to the average δ^18^O_sw_ for an ice-free world^13^, and the equation proposed by Alberti et al.^62^. This equation may only be applied up to 70°^62^. Data from belemnites and ammonites from this study (bivalves excluded as they may represent bottom water conditions), and belemnites from published Jurassic clumped isotope studies. Mid-Jurassic Russian Platform data (upper Callovian to lower Kimmeridgian; *Cylindroteuthis* and *Pachyteuthis* belemnites analysed) from Wierzbowski et al.^38^. Upper Jurassic—Lower Cretaceous Falkland Plateau data (S. hemisphere; *Belemnopsis* belemnites analysed) are from Vickers et al.^39^. For both studies, Δ_47_ data were calculated using the [*Brand*] isotopic parameters^64^ and the temperatures calculated using the Wacker et al.^65^ calibration. Mid-Jurassic Hebrides Basin data (upper Callovian to lower Kimmeridgian; *Cylindroteuthis* and *Pachyteuthis* belemnites analysed) from Vickers et al.^35^ were recalculated to the new I-CDES carbonate-based reference frame^53,54^. Valanginian data (from both northern and southern hemisphere locations; 5 different belemnite genera analysed) from Price et al.^40^. It is not possible to recalculate the older datasets to the new I-CDES reference frame as not enough ETH standards were measured, yet the measured ETH-1 and ETH-3 standards are very close to the I-CDES ETH values, so the data are considered comparable. The different time periods are presented together as the global average will remain -1‰, and the latitudinal δ^18^O_sw_ equation does not take into account palaeogeography. Error bars represent the maximum uncertainty in reconstructed δ^18^O_sw_ by the 95% confidence intervals on the clumped isotope temperatures and the SD of repeat analysis for δ^18^O_bel_.

At Christian Malford (Phaeinum subzone), it happens that the δ^18^O_sw_ average that is calculated for both aragonite and calcite is approximately − 1‰, the average expected ocean value for an ice-free world^13^. When using clumped temperatures and measured δ^18^O carbonate at other sites (Fig. 3 and references therein), we see greater deviations from this global average value, indicating that using a global average value is not representative of all localities, particularly at high and low latitudes and in semi-enclosed basins. Indeed, if another stable isotope equation is used for the belemnite calcites, we still see the large spread in reconstructed δ^18^O_sw_ (as great as 7‰^35,38,40^, Fig. 3). It is clear from the present variation in oxygen isotope composition of surface waters, and from GCM models of δ^18^O_sw_, that applying a global average is unlikely to be representative of a particular location or water depth^14,15^. Alberti et al.^62^ proposed using an empirically derived equation for modern oceans, − 1‰ to account for the absence of Polar ice, to estimate latitudinal δ^18^O_sw_ trends. However, as highlighted by Alberti et al.^62^, this does not take into account paleogeography, therefore it is unlikely to capture local δ^18^O_sw_ variations, particularly for the semi-enclosed basins that characterised the Mesozoic of Europe during the Jurassic (Fig. 1B). Using clumped isotopes temperatures to back-calculate δ^18^O_sw_ reveals that there are large variations in local δ^18^O_sw_ that are not captured using either a global average or empirical gradients based on modern geography (Fig. 3). The studies of Wierzbowski et al.^38^ (Russian Platform) and Vickers et al.^35^ (Hebrides Basin) span broad age ranges and encompass changes in circulation patterns, as reflected in their very large ranges in δ^18^O_sw_ values (Fig. 3). The study of Price et al.^40^ demonstrates that for a single time-slice, latitudinal δ^18^O_sw_ as reconstructed by clumped isotope thermometry does show higher values at the equator than the poles (i.e. more similar to that proposed by Alberti et al.^62^), yet there is a broad and varied spread in the mid-latitudes. In the study of Price et al.^40^ some of the spread may arise from analysing different belemnite genera and species, which may have inhabited different depth habitats. Yet, since large ranges are observed in studies that use only one or two belemnite genera or families^35,38,39^, we believe that a significant portion of the variation arises from local δ^18^O_sw_ fluctuations. Thus, we anticipate that the majority of published values are still likely to under- or over-estimate local δ^18^O_sw_, leading to an over- or under-estimation in seawater temperatures, even if using the Kele et al.^27^ equation to calculate sea temperatures.

To conclude, we provide the first seawater Δ_47_ temperatures for the Jurassic based on samples that most likely have not been modified by diagenetic processes nor by ^13^C-^18^O bond reordering. We show that the palaeothermometry equations that have traditionally been applied to belemnite calcite are inappropriate, and, therefore, that reconstructions based on them grossly underestimate palaeotemperatures for the Mesozoic. The present study implies that ca. 11–12 °C needs to be added to the temperature estimates for the Jurassic and Cretaceous that were based on belemnites^4–12^ taking the average δ^18^O_calcite_ of combined belemnite datasets and the 2 standard deviation range^12^. This has substantial implications for our understanding of the Mesozoic world and Greenhouse Earth-system states. It raises some of the lowest temperature estimates for the Jurassic period (such as the Bajocian “cold mode”; ~ 7 °C) to values which approach modern mid-latitude sea surface temperatures, thereby undermining previously speculated “icehouse” phases^9^. The warm belemnite temperatures do not contradict cooler temperature estimates from co-occurring benthic organisms; rather, they may be used to understand vertical temperature profiles in the oceans.

## Methods

The carbonate material used in this study all comes from a 2 m thick interval in the Callovian-aged Peterborough Member of the Oxford Clay Formation (Athleta Zone, Phaeinum subzone). The Peterborough Member consists of alternating organic-poor, shell-rich massive clay and organic-rich, variably shelly, fissile clay^42,66^ All material was collected from an excavation site at Christian Malford, Wiltshire, U.K. (Fig. 1). Published ICP-MS, SEM and CL work on selected *Cylindroteuthis* belemnite aragonite and calcite material indicates the exceptional quality of preservation of these carbonates^42^, as does the PXRD, ICP-OES, SEM, EBSD and EDS element maps presented in this study (Supplementary material Figs. S2, S3, S4, S5, S6, S7, S8, S9, S10, S11, S12). Estimates suggest a maximum burial depth of only c. 500 metres^45^, indicating that significant post-depositional heating did not occur. Due to the limited amount of some of the aragonitic material (particularly phragmocones) it was not possible to analyse all samples for ICP-OES, SEM and PXRD, yet it is assumed that the analysed samples are representative for all samples used in this study because minimal variability was observed.

### PXRD

Powder X-ray diffraction (PXRD) was carried out using a Stoe StadiP transmission (capillary) diffractometer with a copper anode at 30 mA, 40 kV and a germanium 111 monochromator to produce Kα1 X-rays. The diffracted beam was collected by an 18° 2θ Dectris Mythen1K silicon strip detector. Samples were loaded in 0.3 mm borosilicate glass capillaries, mounted and aligned on the goniometer head and set to spin continuously during data collection. Both data sets were scanned from 10 to 55° 2θ stepping at 0.5° and 5 s/step. The resultant raw data has a step of 0.015° 2θ. Machine alignment was monitored using an NBS silicon standard. Phase analysis was done using Bruker’s “Eva” program^67^ interfaced with the Powder Diffraction File provided by the International Centre for Diffraction Data.

### Microscopy

Scanning electron microscopy was undertaken on selected samples, in order to assess whether the original biomineral crystal habits are preserved, and to identify the best-preserved regions within the belemnite rostra. Analyses were performed using secondary electrons on an FEI Quanta Inspect 250 Scanning Electron Microscope under a high vacuum of 2.40 to 2.93·10^–4^ Pa and an electron beam of 95–97 μA at the Geological Museum in Copenhagen, out on selected aragonites from phragmocones, ammonites and bivalves analysed in this study.

For SEM–EDS and EBSD analysis, cross- and longitudinal- sections of a selected rostra were mounted in epoxy and mechanically polished down to a 0.25 µm diamond solution grain size, followed by chemical–mechanical polishing using an alkaline solution of colloidal silica in a neoprene substrate. EBSD orientation mapping was performed on the coated sample (~ 2.5 nm carbon) in a Thermo Fischer—FEI Quanta 200F equipped with an EDAX Hikari EBSD camera and TEAM software for data acquisition at the Scientific Center for Optical and Electron Microscopy (ScopeM) at ETH Zurich. Acquisition was performed with an accelerating voltage of 20 kV, beam current of 8 nA, working distance of 17 mm, and mapping step size of 1 µm. Post-acquisition cleaning included grain confidence index (CI) standardization followed by one step of grain CI correlation. All points with CI < 0.1 and grains with less than 10 pixels were removed to prevent artifacts in the calculations.

The SEM–EDS element maps were undertaken at the SEM laboratory at the Geological Survey of Denmark and Greenland (GEUS), which hosts a ZEISS sigma 300VP field emission scanning electron microscope that is equipped with 2 Bruker Xflash 6|30 129 eV EDS detectors and a Bruker e-Flash FS EBSD detector. Element maps were acquired from infilled apical area to the outermost pyritised rim of the rostrum, covering the changes of growth ring density and mineralogical changes. The sets of transect section were obtained for the cross- and the longitudinal-section. Elements mapped (Ca, Mg, Fe, Mn, O) cover the range of possible carbonates, with aragonite distinguished by trace Sr; the potential occurrence of apatite (P), pyrite (Fe, S), clays (Al, Si, Ba, K) and quartz (Si) was also tested.

### ICP-OES

Minor element analyses were performed using an Agilent 5110 VDV ICP-OES at the Camborne School of Mines, University of Exeter, following methods laid out in detail in Ullmann et al.^68^. The minor element data are expressed as ratios to Ca. Fossil samples were dissolved in 2% v/v HNO_3_ with a dilution factor of ~ 16,000, yielding a nominal Ca concentration of 25 µg/g in solution. Signal quantification was carried out using a four point calibration using a blank solution and three matrix matched calibration solutions made up from certified single element standards mixed to match the chemical composition of the analysed samples. Accuracy and precision of the analyses was controlled by multiple measurements of interspersed international reference materials (JLs-1 and AK) and a quality control solution (BCQ2). Repeatability of the measurements is generally better than 1% (2 relative standard deviations) unless affected by limited count rates (el/Ca < c. 100 times the quantification limit). Repeatability of element/Ca ratios in the latter case are 1 µmol/mol for Mn/Ca, 3 µmol/mol for Fe/Ca, 0.3 mmol/mol for S/Ca and 0.2 mmol/mol for P/Ca. Quantification limits for the measurements computed as six times the standard deviation of the measurement blank are 9 µmol/mol for Mg/Ca, 0.2 µmol/mol for Sr/Ca, 1 µmol/mol for Mn/Ca, 4 µmol/mol for Fe/Ca, 0.4 mmol/mol for S/Ca and 0.2 mmol/mol for P/Ca. Due to the generally limited quantity of phragmocone material available, it was not possible to analyse all phragmocone samples for ICP-OES. ICP-OES data are reported and plotted in the Supplementary Material.

### Clumped isotope thermometry

Powdered homogenized rostrum samples were collected from across the middle chambers (apex and outer edge avoided), away from the tip of the rostrum, using a Dremel drill. For the aragonitic material (phragmocones and ammonites), small pieces were picked off using tweezers, and powdered using an agate mortar and pestle. With the phragmocones, it was not possible to select specific chambers or homogenize across many chambers, due to limited aragonitic belemnite material remaining.

Clumped isotope measurements were carried out at the ETH Zurich using a ThermoFisher Scientific MAT253 mass spectrometer coupled to a Kiel IV carbonate preparation device, following the methods described in Müller et al.^69^. The Kiel IV device included a PoraPakQ trap kept at -40 °C to eliminate potential organic contaminants. Samples were measured between May 2019 and December 2020 by measuring maximum 3 replicates of each sample per run which consists generally of 24 samples of 130–150 µg interspersed with 20 replicates of each of the three carbonate standards ETH-1, ETH-2 and ETH-3^70^. The samples were analysed in LIDI mode with 400 s of integration of sample and reference gas. The calculations and corrections were done with the software Easotope^71^ using the revised “Brand parameters” for ^17^O correction as suggested by Daëron et al.^64^. The data are reported with respect to the Intercarb carbon dioxide equilibration scale (I-CDES)^53^. Temperatures were calculated using the Anderson et al.^54^ calibration which is based on the re-measurement of a series of samples that were used for previous calibrations. Their consistency with calibrations based on biogenic carbonates^72–74^ suggest that it is appropriate for marine biogenic carbonates. Corrections for aragonite were the same as for calcite except that we used the phosphoric acid oxygen isotope correction for aragonite of 1.00909^75^.

## Supplementary Information

Supplementary Information 1.

Supplementary Information 2.
