# Supplementary material for: Marine temperatures underestimated for past greenhouse climate
Source: Sci Rep. 2021 Sep 27;11:19109. doi: 10.1038/s41598-021-98528-1 (PMC8476565; doi:10.1038/s41598-021-98528-1)
Supplement: Supplementary file 1 — Supplementary Information 1. [file 41598_2021_98528_MOESM1_ESM.pdf]

Supplementary Figures

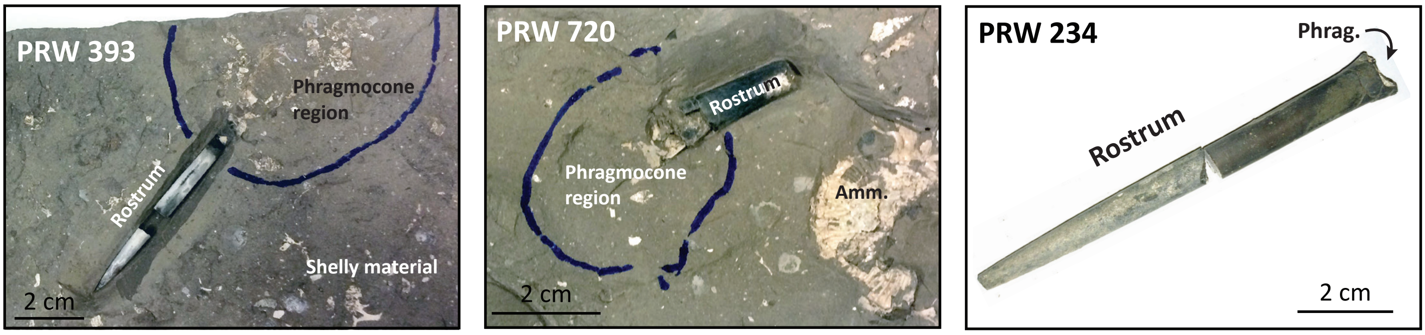

Supplementary Figure S1: Photographs of selected samples used in this study, showing both biogenic aragonite and calcite. Amm. = ammonite; Phrag. = phragmocone.
Photographer: M.L. Vickers

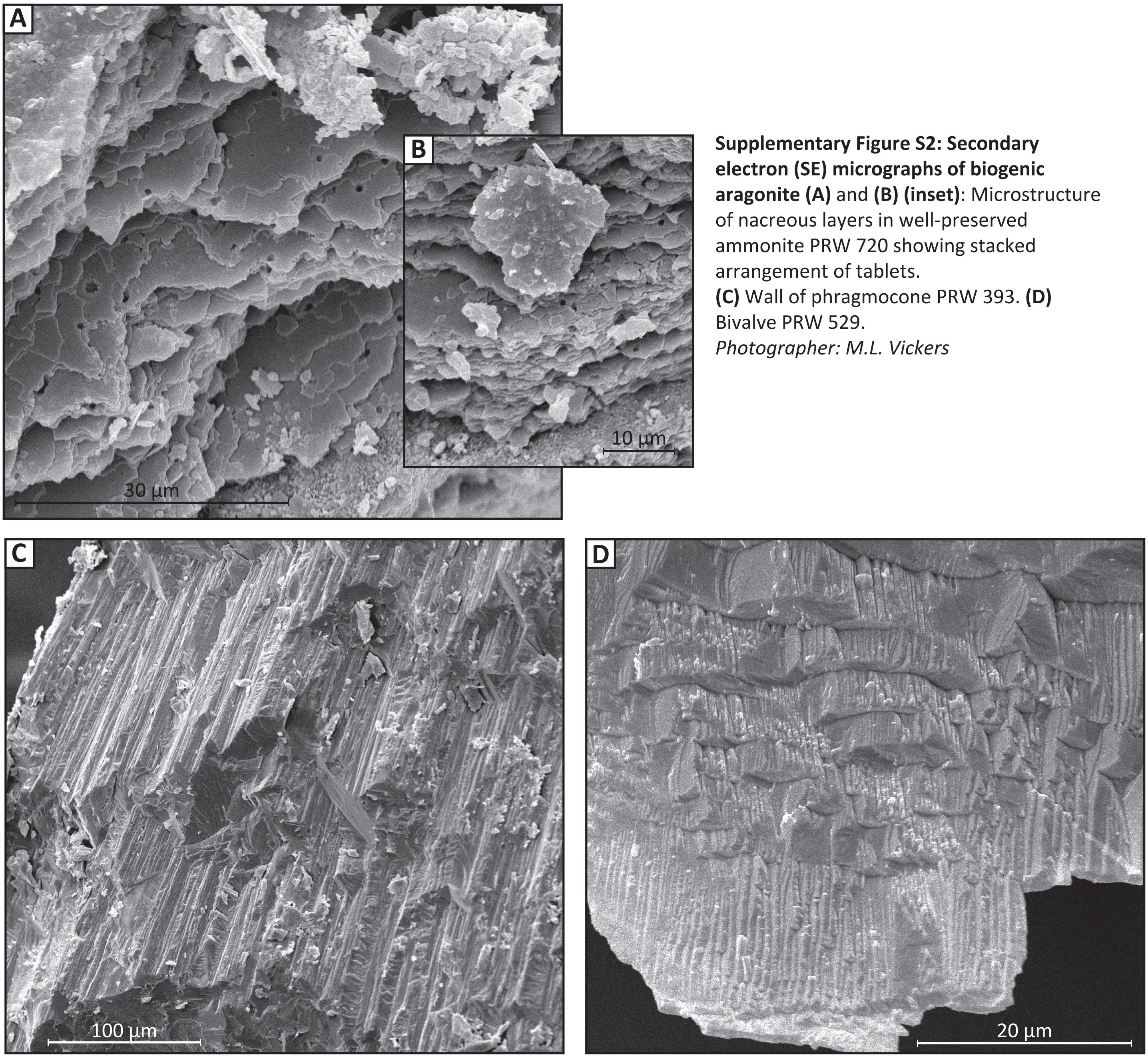

Supplementary Figure S2: Secondary electron (SE) micrographs of biogenic aragonite (A) and **(B) (inset)**: Microstructure of nacreous layers in well-preserved ammonite PRW 720 showing stacked arrangement of tablets.
(C) Wall of phragmocone PRW 393. **(D)** Bivalve PRW 529.
Photographer: M.L. Vickers

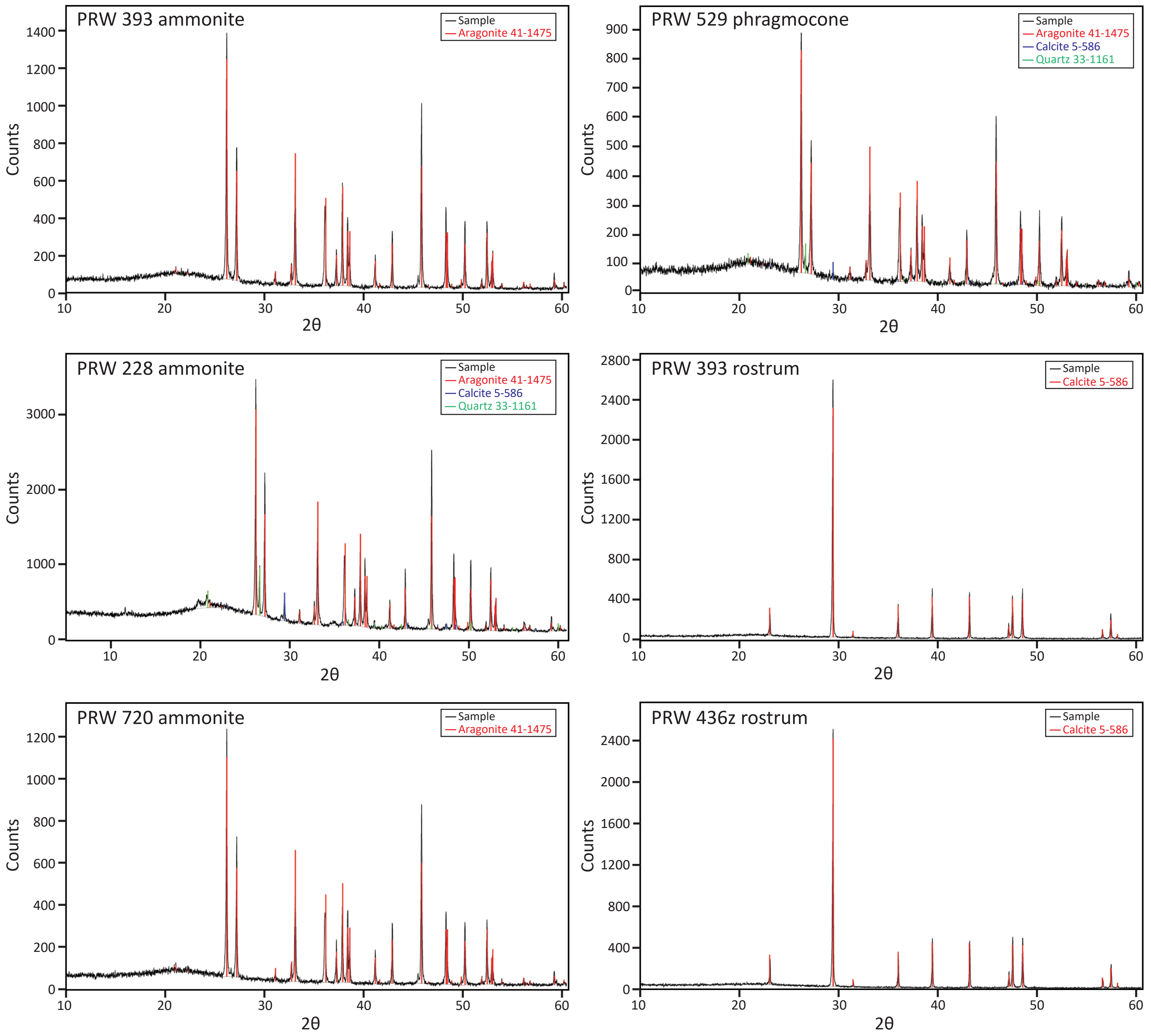

Supplementary Figure S3: Powder X-Ray Diffraction traces for a subsample of the biogenic samples used in this study. Due to the limited amount of aragonitic material, there was some contamination in some samples from the host sediments, accounting for the small amount of quartz observed in PRW 228 (ammonite) and PRW 529 (phragmocone). Furthermore, it was only possible to analyse aragonitic material from one belemnite due to the very limited phragmocone material available. As this and the ammonites are all aragonitic, it is believed that these are representative of all the biogenic aragonite, and therefore we can assume that all phragmocone material analysed for clumped isotopes is aragonitic.

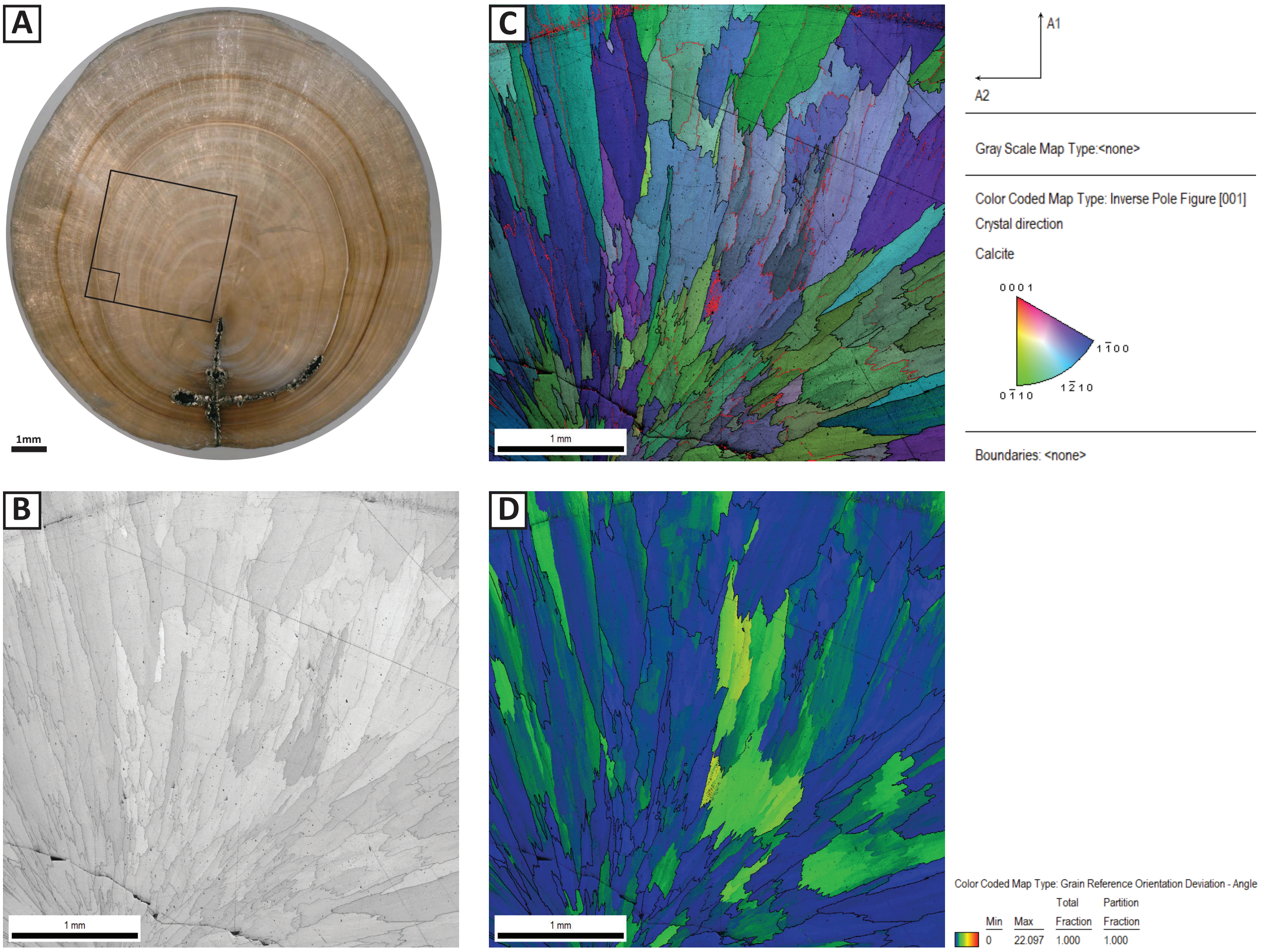

Supplementary Figure S4: (A) Cross-section of a belemnite rostrum (PRW 363) with the black square showing the location and orientation of the electron backscatter diffraction (EBSD) maps as presented in the figures B-D. **(B)** Image quality map derived from the quality of the diffraction patterns for every pixel in the map, where the better the quality of the diffraction pattern, the brighter the pixel. **(C)** EBSD orientation map with inverse pole figure (IPF) color-coding combined with image quality map. In this color-code, one selects a given axis in the map (in this case, normal to the screen) and the color of the grains are given according to the inset in (C). In this case, if a grain is dark blue as in the corner of the triangle, that means that the normal to the (1-100) plane is pointing to the reader, if a grain is light blue, the normal to the (1-210) is pointing to the reader, and so on. That implies that the calcite fibers in this map are roughly elongated along its [0001] axis. The grain boundaries marked in black are the boundaries with misorientation varying from 10° to 110°, while in red are low angle grain boundaries, with angles varying from 2° to 10°. **(D)** Grain reference orientation deviation map, which shows the misorientation between the orientation of every pixel in its respective position and the reference or mean orientation of the grain. This map gives an idea of the lattice distortion of the calcite crystals in degrees, from virtually undistorted crystals (blue) to mildly distorted (green-yellow) to strongly distorted grains (red). Note that the grains with higher distortion tend to be slightly darker in the image quality map in (B), which is one of the factors that influence diffraction pattern quality.

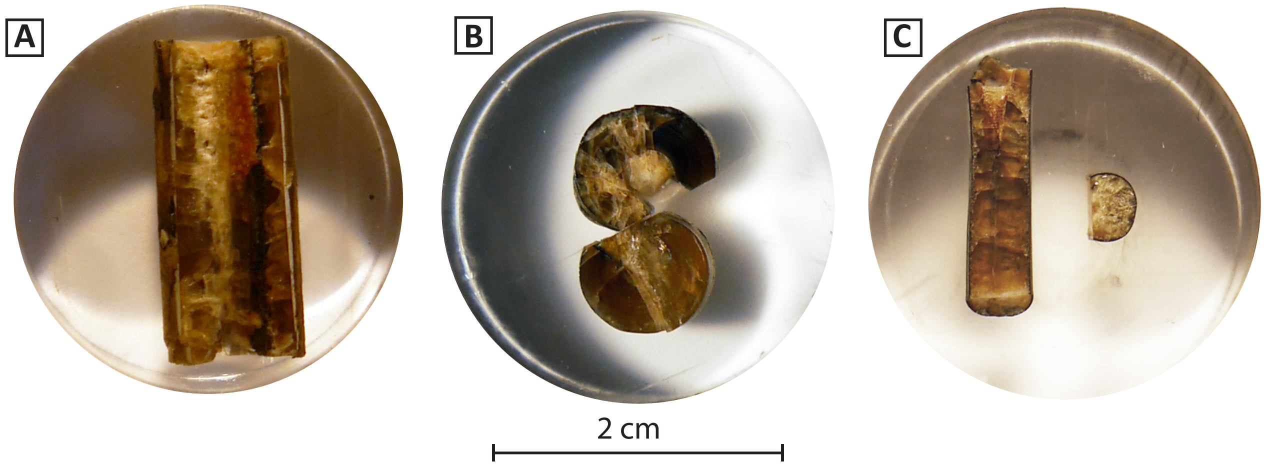

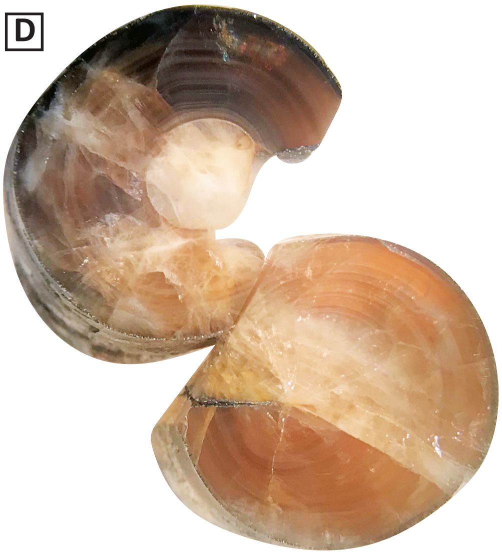

Supplementary Figure S5: Polished belemnite thick sections.(A) Longitudinal section of belemnite rostrum PRW 234. **(B)** Cross sections through the tip and phragmocone ends of rostrum PRW 234. **(C)** Longitudinal and cross section of belemnite PRW 393. **(D)** Detail of cross sections of PRW 234.**(E)** Detail of phragmocone and rostra of PRW 393. *Photographer: S. Lode*

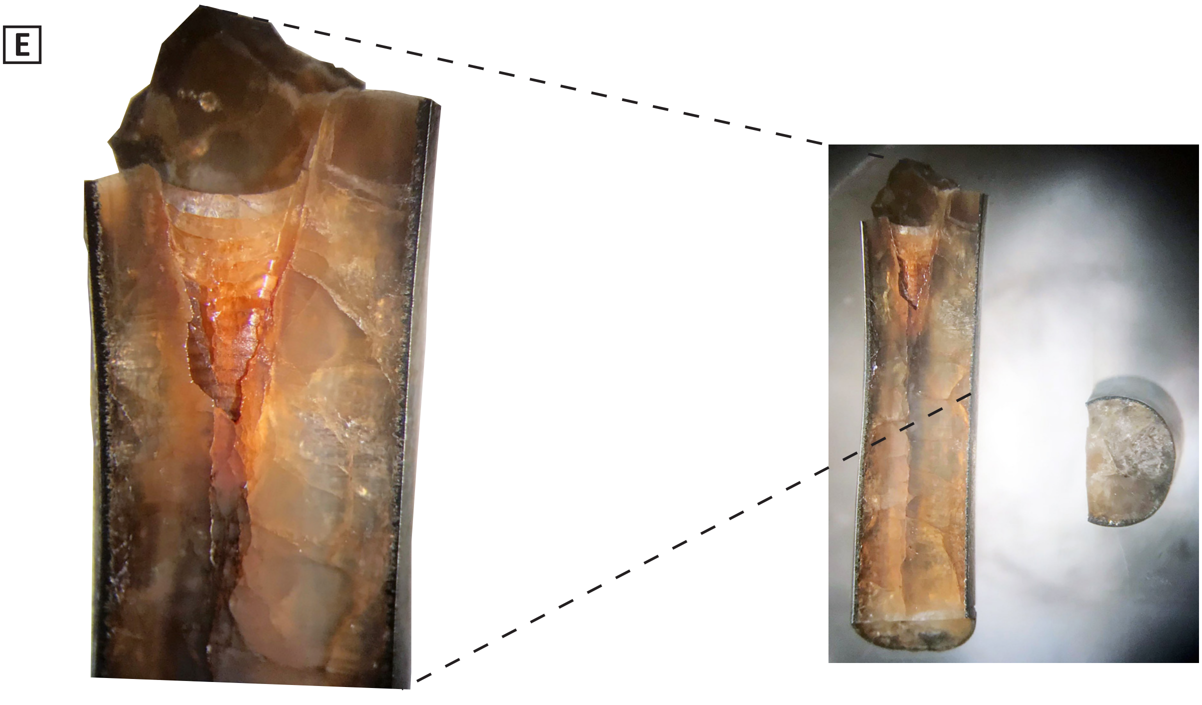

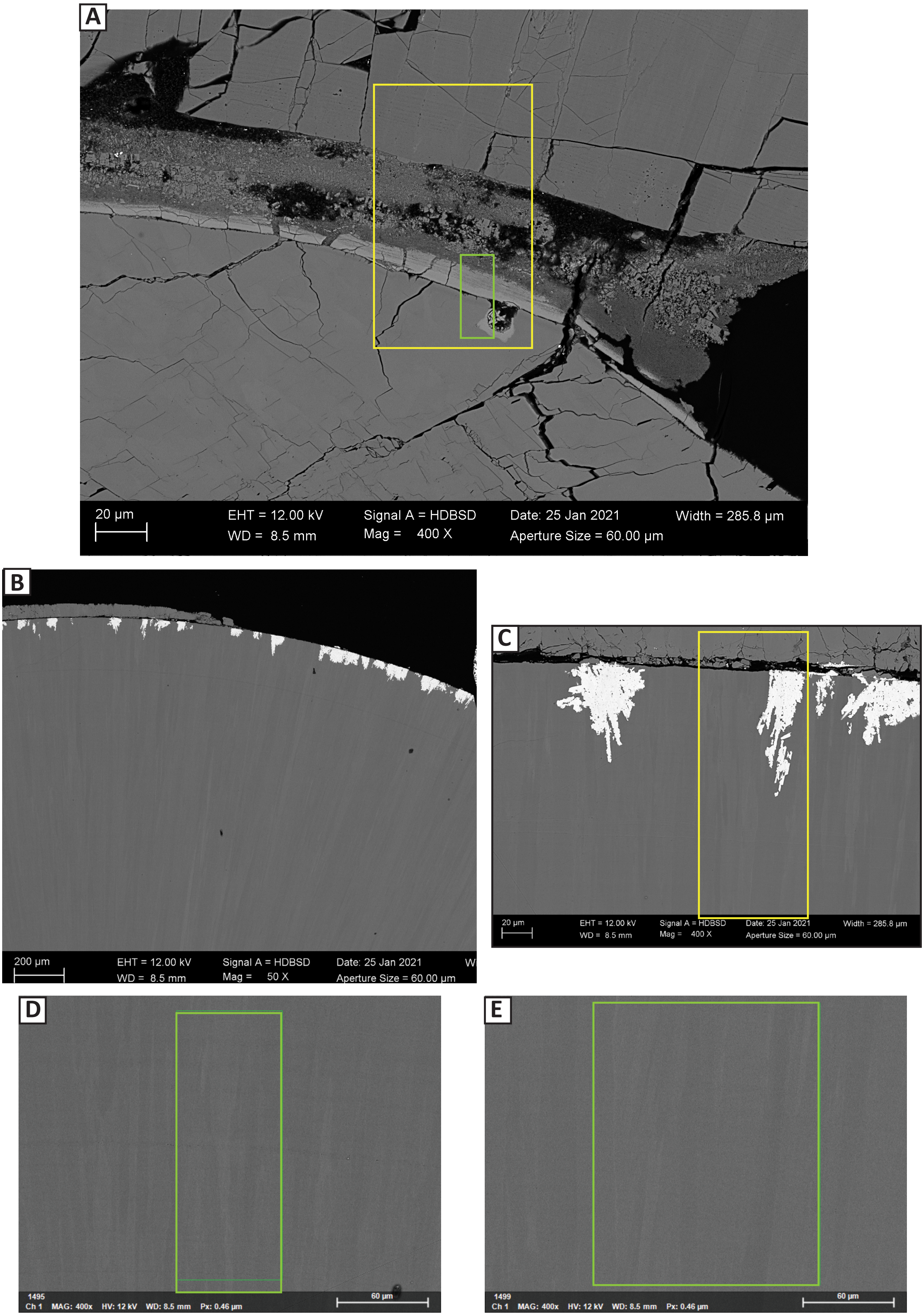

Supplementary Figure S6: BSE SEM photomicrographs of belemnite sample PRW 393

(A) Longitudinal section of PRW393, at the contact between the phragmocone and rostrum. Yellow and green rectangles denote EDS-mapped areas (S7 and S8, respectively) **(B)** Detail of the rim of the cross section of PRW234, showing pyrite infilling the bored edge. **(C)** Enlarged view of pyritised rim of PRW393. Yellow rectangle denotes EDS mapped area (S9). **(D)** & **(E)** Enlarged views of well-preserved area of PRW234 cross-section. Green rectangles denote areas of element maps shown in S10 & S11.

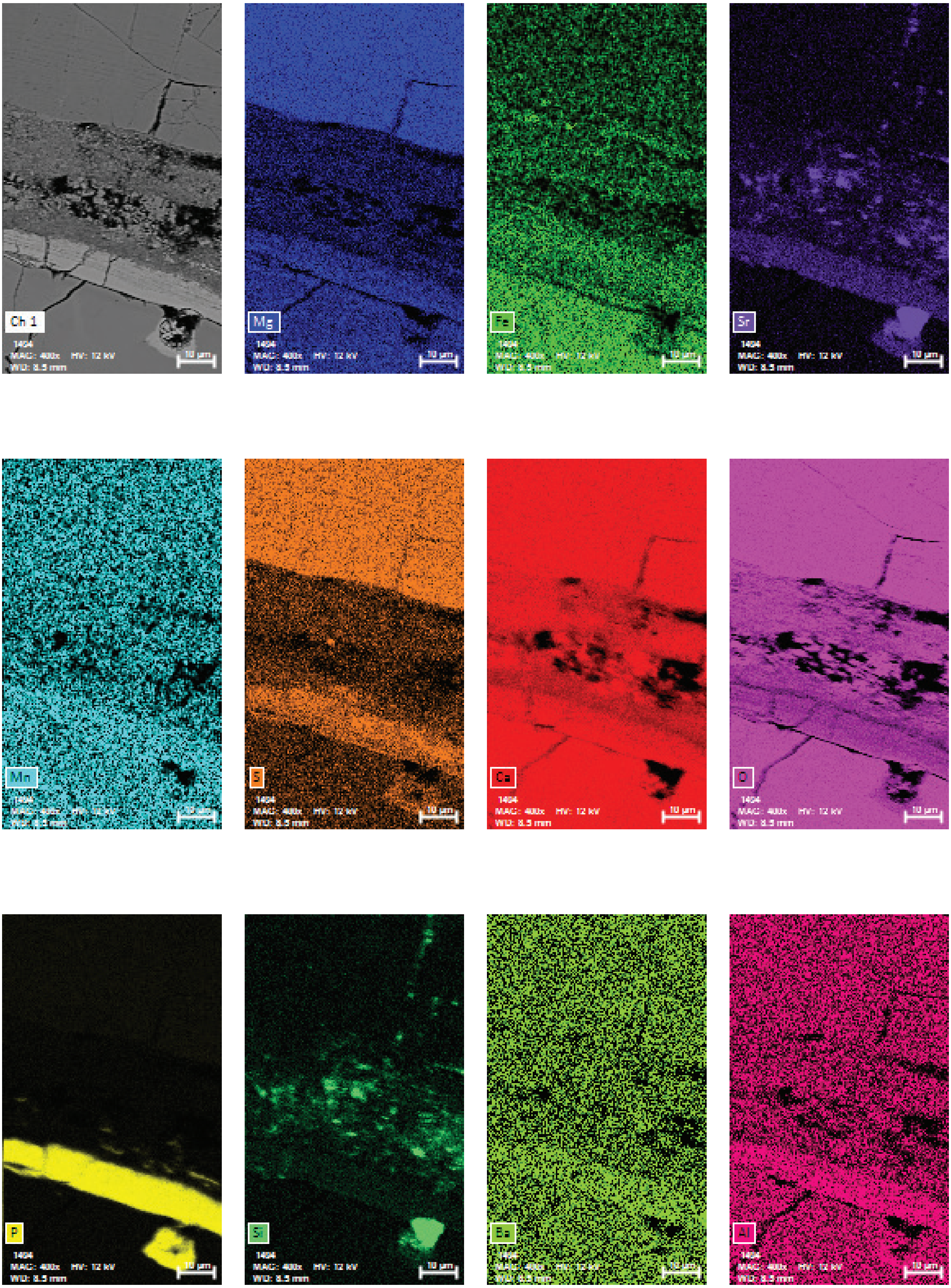

Supplementary Figure S7: EDS element maps of area indicated in S6A by yellow rectangle on the longitudinal section of belemnite PRW 393.

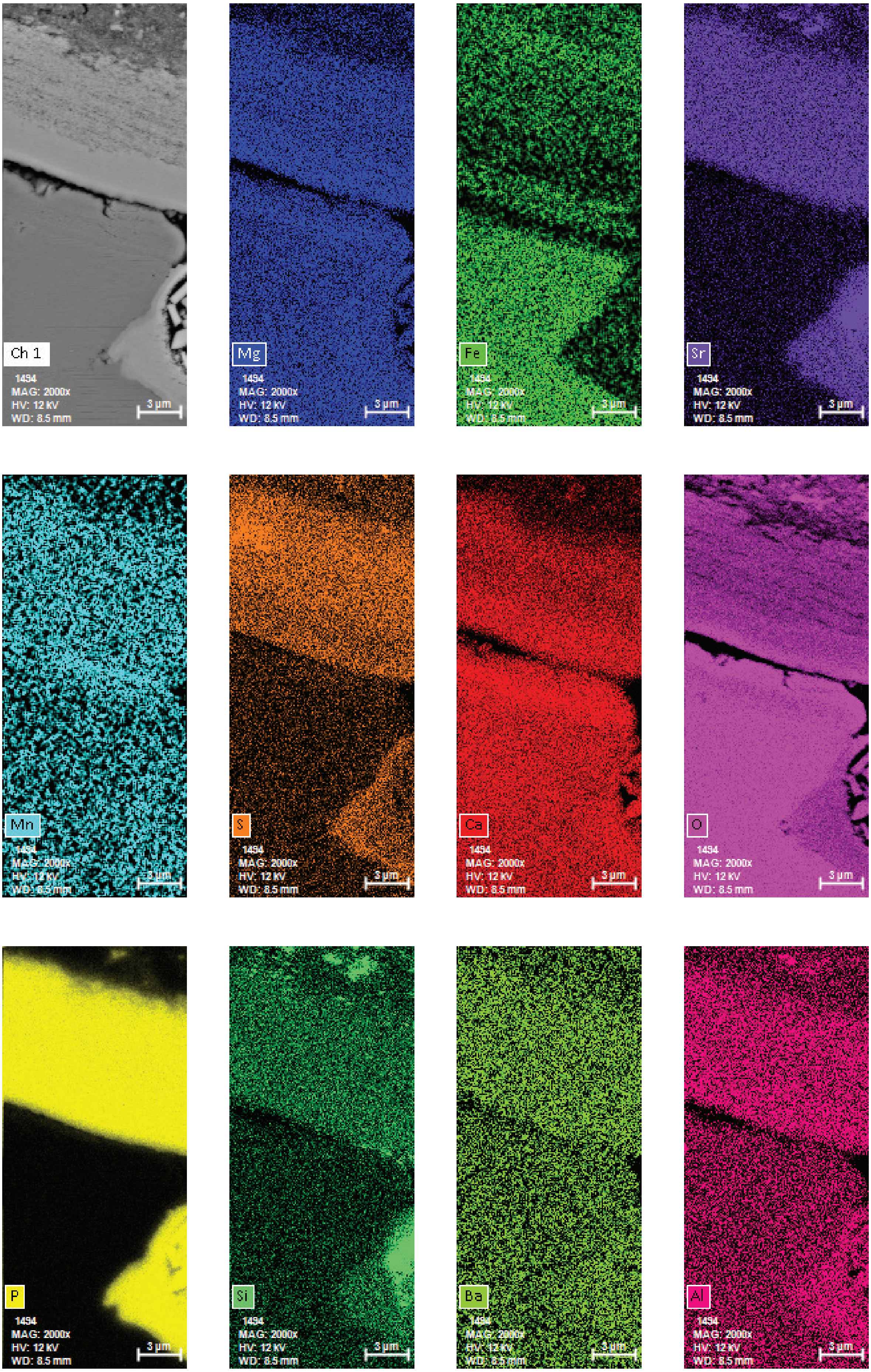

Supplementary Figure S8: EDS element maps of area indicated in S6B by green rectangle on the longitudinal section of belemnite PRW 393.

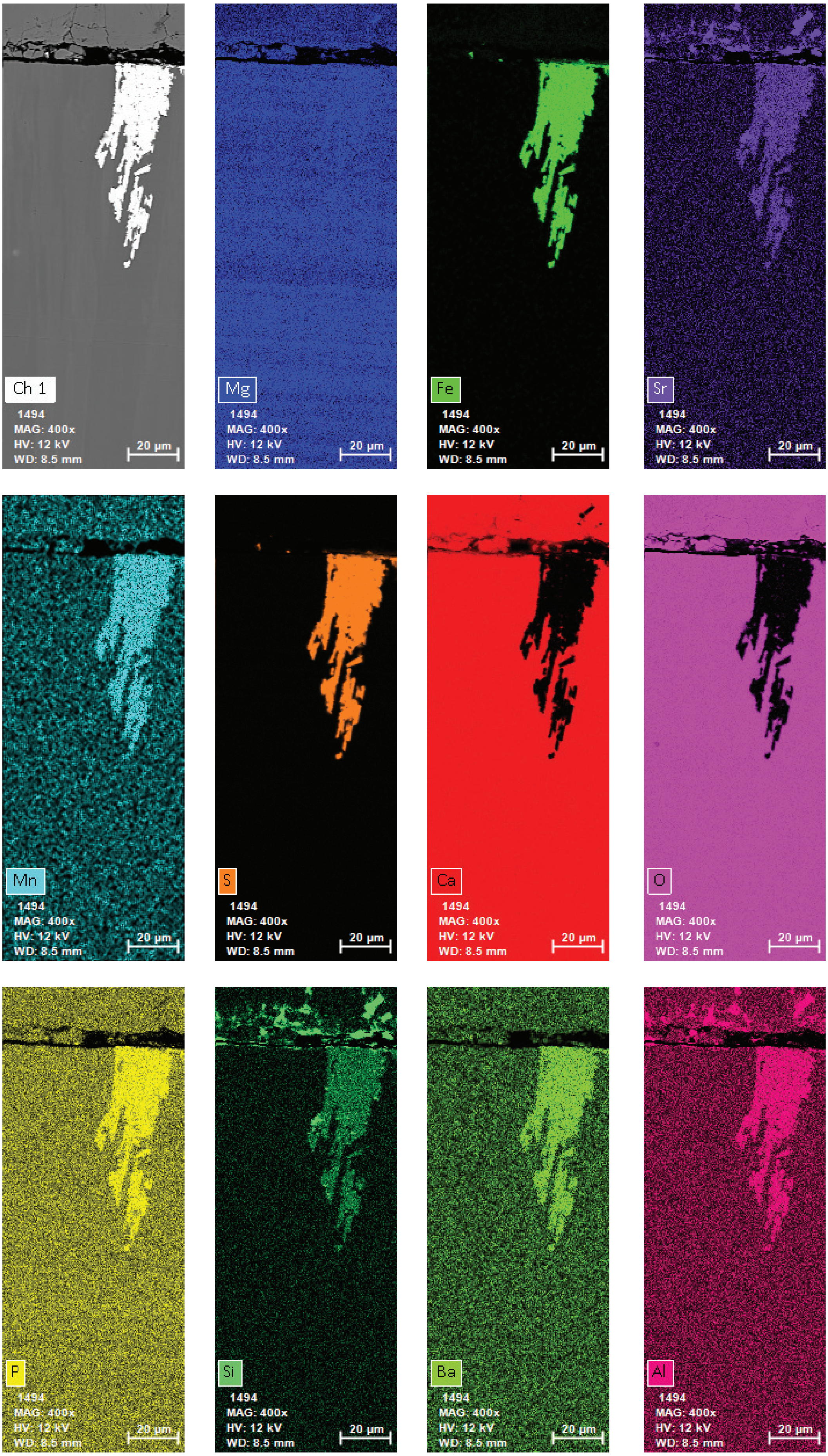

Supplementary Figure S9: EDS element maps of area indicated in S6C by yellow rectangle on the cross-section of belemnite PRW 234.

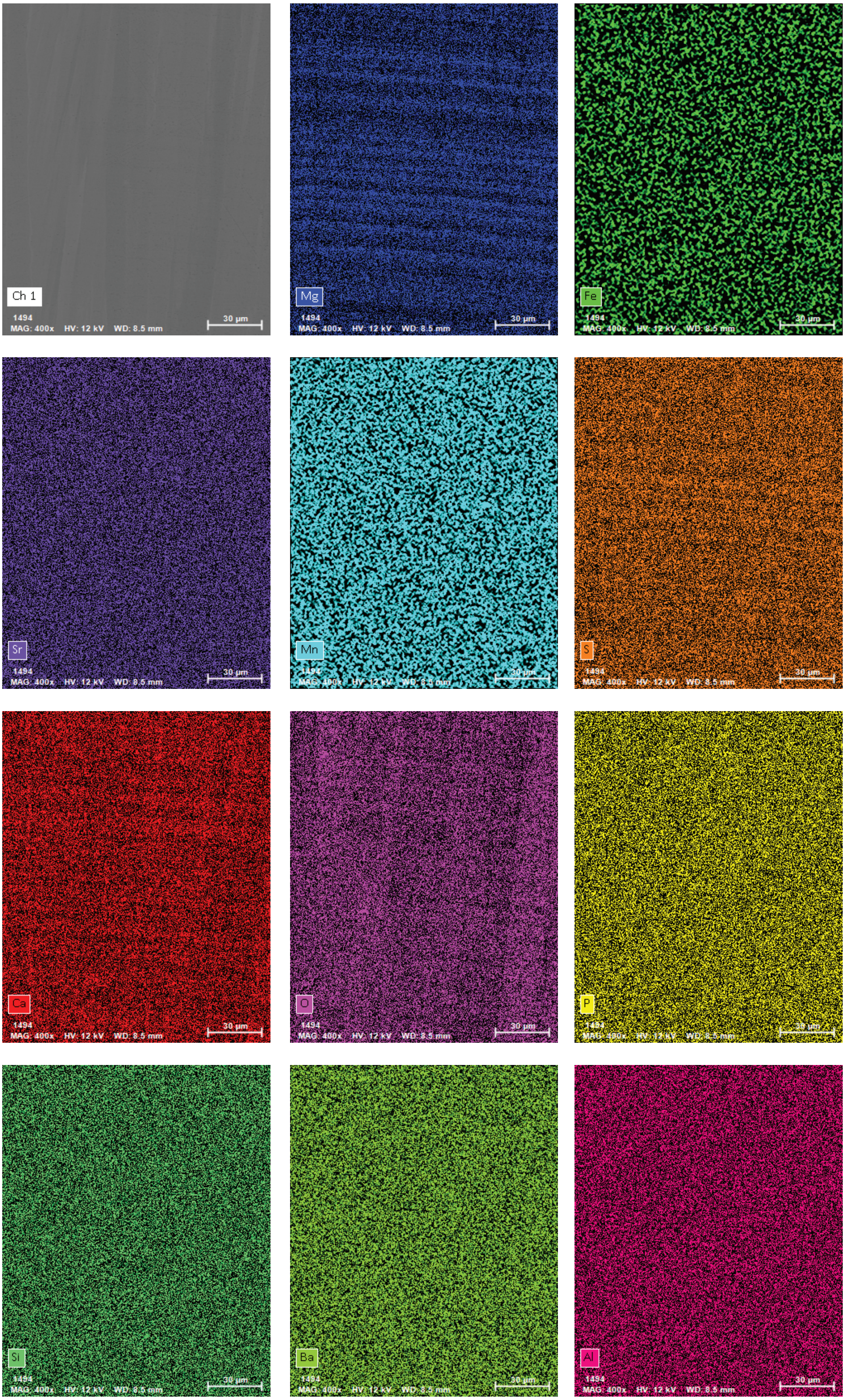

Supplementary Figure S10: EDS element maps of area indicated in S6E by green rectangle on the cross-section of belemnite PRW 234.

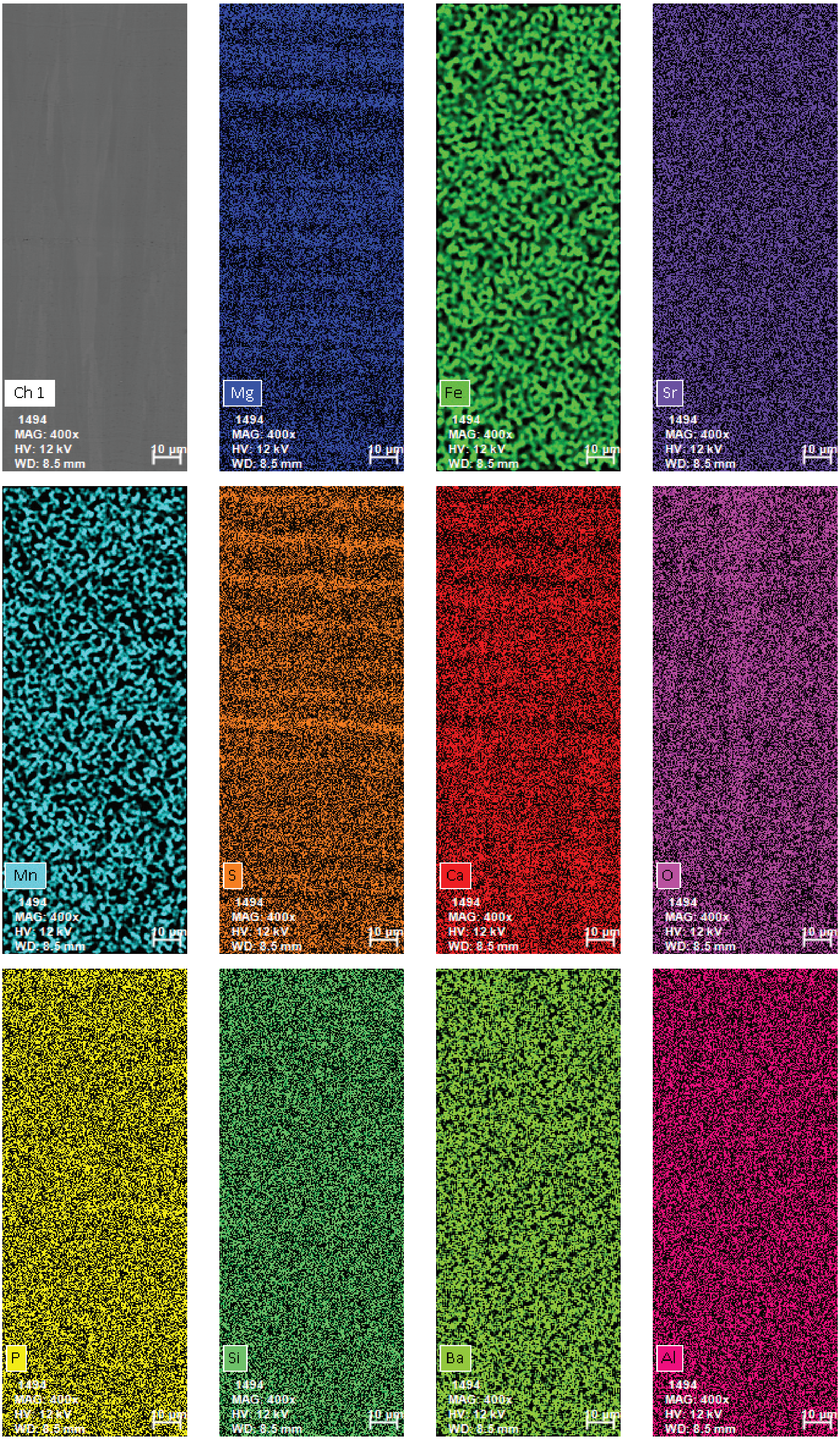

Supplementary Figure S11: EDS element maps of area indicated in S6D by green rectangle on the cross-section of belemnite PRW 234.

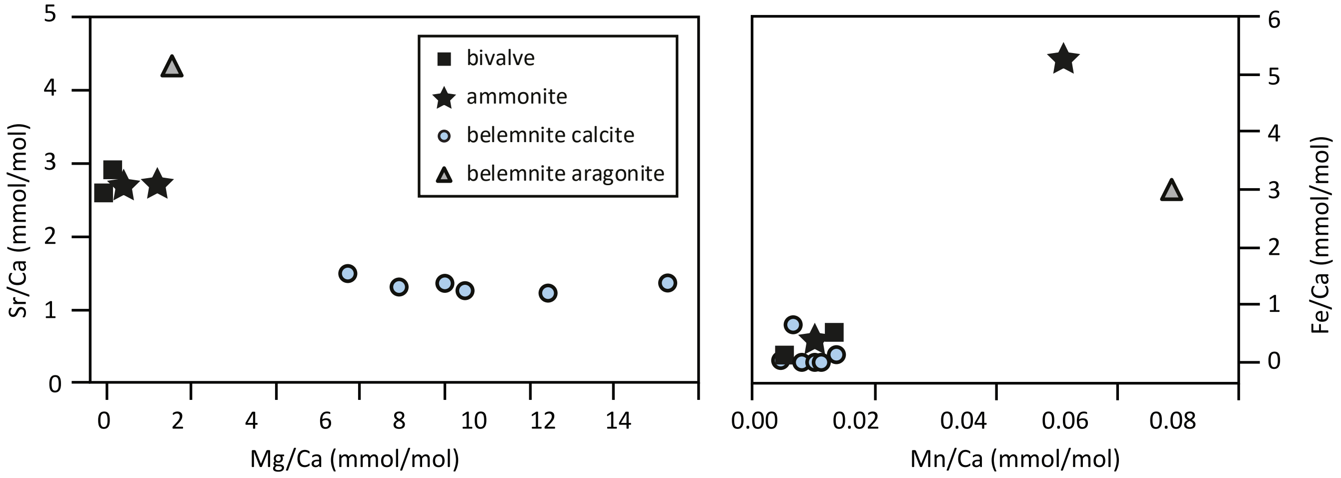

Supplementary Figure S12: ICP-OES-derived minor element ratios for the analysed samples.

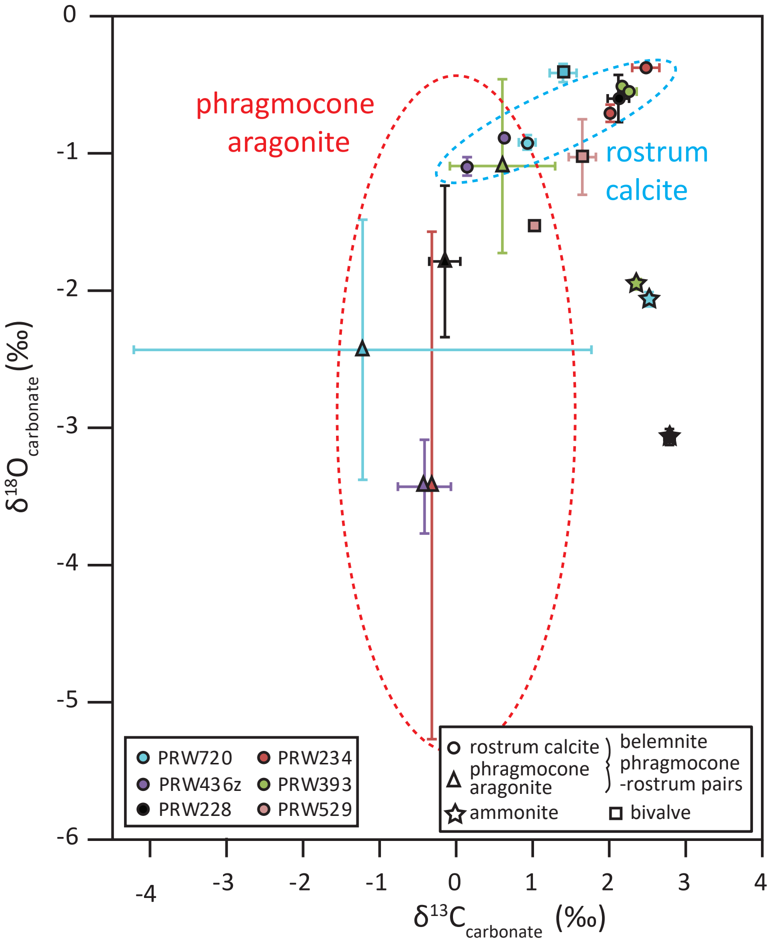

Supplementary Figure S13: Stable isotope ratios for the analysed carbonates. Phragmocone – rostrum pairs and samples from the same block indicated by colour.

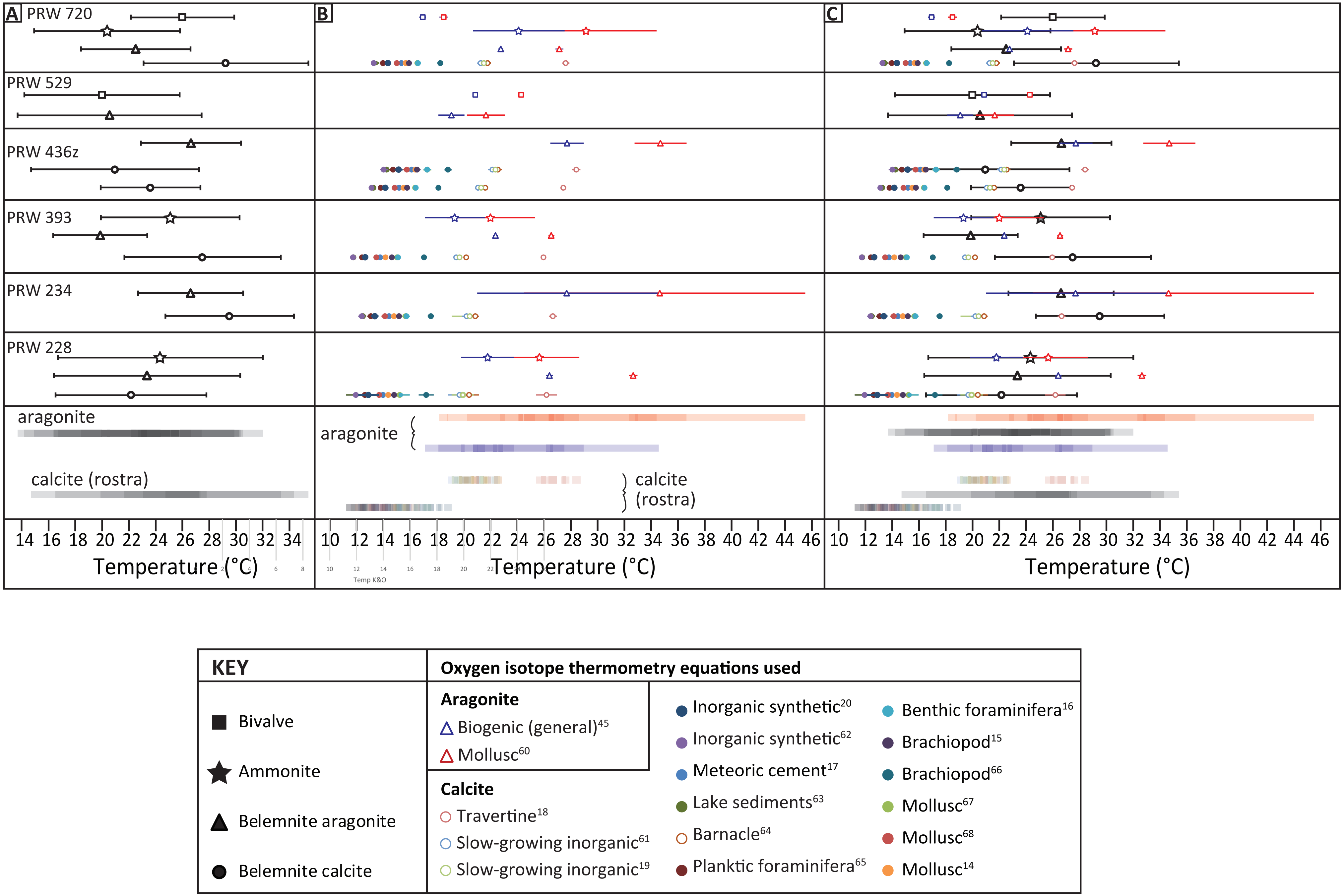
A
B
C
PRW 720
PRW 529
PRW 436z
PRW 393
PRW 234
PRW 228
aragonite
calcite (rostra)
14 16 18 20 22 24 26 28 30 32 34
10 12 14 16 18 20 22 24 26 28 30 32 34 36 38 40 42 44 46
Temperature (°C)
Temp K&O
KEY
Bivalve
Ammonite
Belemnite aragonite
Belemnite calcite
Oxygen isotope thermometry equations used
Aragonite
Biogenic (general)[45]
Mollusc[60]
Calcite
Travertine[18]
Slow-growing inorganic[61]
Slow-growing inorganic[19]
Inorganic synthetic[20]
Inorganic synthetic[62]
Meteoric cement[17]
Lake sediments[63]
Barnacle[64]
Planktic foraminifera[65]
Benthic foraminifera[16]
Brachiopod[15]
Brachiopod[66]
Mollusc[67]
Mollusc[68]
Mollusc[14]

Supplementary Figure S14 (previous page): (A) Reconstructed temperatures from clumped isotopes, based on 10 or more replicates, for all samples analysed (all from the Phaeinum subzone), separated by type (rostra, phragmocone, ammonite and bivalve) and placed in sections for samples from the same block. The bottom plot groups all samples together to demonstrate the range spanned over this single subzone. Error bars indicate the 95% confidence interval. **(B)** Reconstructed oxygen isotope temperatures using a wide range of published calcite and aragonite (see key). An assumed $\delta^{18}O_{sw}$ of -1 ‰ was used in each case. **(C)** Measured clumped isotope temperatures overlaid by reconstructed oxygen isotope temperatures.

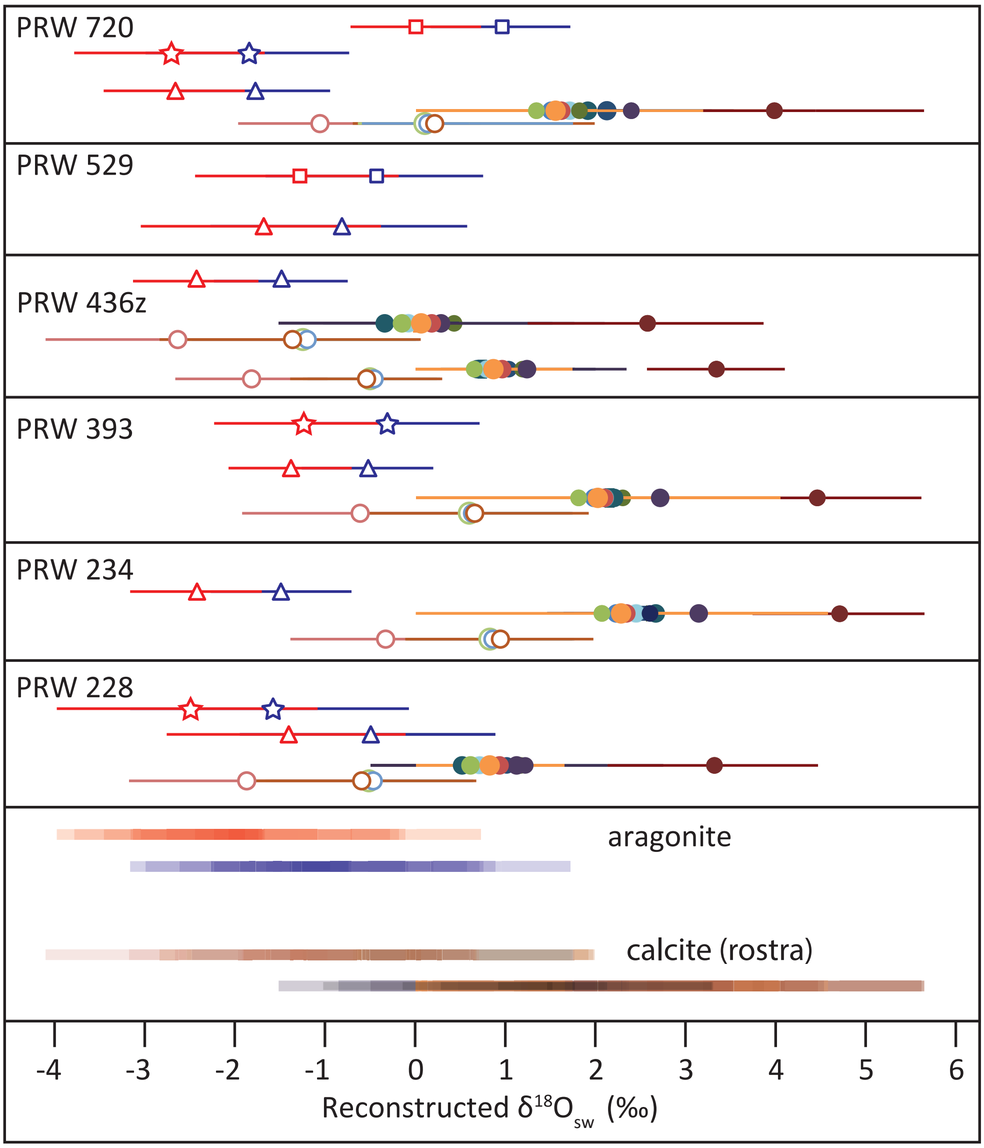

Supplementary Figure S15: Reconstructed $\delta^{18}O_{sw}$ using the measured clumped isotope temperatures and the oxygen isotope thermometry equations listed in Supp. Fig. S14.
